# Estimated Prevalence of Smoking and Smoking-Attributable Mortality Associated With Graphic Health Warnings on Cigarette Packages in the US From 2022 to 2100

**DOI:** 10.1001/jamahealthforum.2021.2852

**Published:** 2021-09-24

**Authors:** Jamie Tam, Jihyoun Jeon, James F. Thrasher, David Hammond, Theodore R. Holford, David T. Levy, Rafael Meza

**Affiliations:** 1 Department of Health Policy and Management, Yale University School of Public Health, New Haven, Connecticut; 2 Department of Epidemiology, University of Michigan School of Public Health, Ann Arbor; 3 Department of Health Promotion, Education, and Behavior, Arnold School of Public Health, University of South Carolina, Columbia; 4 School of Public Health and Health Systems, University of Waterloo, Waterloo, Ontario, Canada; 5 Department of Biostatistics, Yale University School of Public Health, New Haven, Connecticut; 6 Lombardi Comprehensive Cancer Center, Georgetown University Medical Center, Washington, DC

## Abstract

**Importance:**

Starting in 2022, the US Food and Drug Administration (FDA) plans to require all cigarette packages in the US to display graphic health warnings depicting health harms associated with smoking. The FDA originally planned to implement such warnings in 2012, but tobacco industry litigation delayed the effort.

**Objective:**

To assess the estimated population health outcomes associated with a policy requiring graphic health warnings on cigarette packages in the US and with a 10-year delay in implementation.

**Design, Setting, and Participants:**

This decision analytical model used simulation modeling of smoking prevalence and smoking-attributable mortality in the US from 2012 to 2100, using the Cancer Intervention and Surveillance Modeling Network smoking population model. The study was conducted from October 2020 to July 2021.

**Main Outcomes and Measures:**

The primary outcomes were annual adult smoking prevalence, smoking-attributable deaths averted, and life-years gained vs the baseline scenario. A baseline scenario assuming no graphic health warnings was compared with expected outcomes of implementing graphic health warnings in 2022 vs 2012. Policy effects were considered under varying assumptions of the association of the policy with smoking initiation and cessation, ranging from most conservative to most optimistic. A maximum smoking reduction scenario in which all smoking would stop by the end of 2022 was evaluated.

**Results:**

In the baseline scenario, an estimated 13.2 million smoking-attributable deaths would have occurred from 2012 to 2100. Under a maximum smoking-reduction scenario, 5.5 million of these deaths would be averted. Implementation of graphic health warnings from 2022 to 2100 would be associated with 539 000 (range, 275 000-794 000) smoking-attributable deaths averted and 7.9 million (range, 4.0-11.6 million) life-years gained, representing less than 10% of the 5.5 million estimated smoking-attributable deaths averted and 81.8 million life-years gained in the maximum smoking-reduction scenario. Implementation from 2012 to 2100 would be associated with 33.2% (range, 32.9%-33.5%) more deaths averted (718 000; range, 365 000 to 1.1 million) and 42.7% (range, 42.3%-43.1%) more life-years gained (11.2 million; range, 5.7-16.6 million) compared with implementation in 2022.

**Conclusions and Relevance:**

This decision analytical model estimated that FDA cigarette graphic health warnings, if implemented in 2022, would be associated with public health benefits. The model also estimated that more smoking-attributable deaths would have been averted if the policy had been implemented in 2012. Industry litigation and delays to implementing tobacco regulations may have been harmful for public health.

## Key Points

**Question:**

What are the estimated population health outcomes associated with implementation of graphic health warnings on cigarette packages in 2022 and with delayed implementation of the warnings since 2012, as originally planned, in the US?

**Findings:**

In this decision analytical model using simulation modeling of smoking prevalence and smoking-attributable mortality in the US from 2012 to 2100, graphic health warnings, if implemented from 2022 to 2100, would be associated with 539 000 smoking-attributable deaths averted and 7.9 million life-years gained, and if implemented in 2012, with 718 000 deaths averted and 11.2 million life-years gained through 2100.

**Meaning:**

These findings suggest that graphic health warnings on cigarette packages may be associated with important public health benefits, and industry litigation and procedural delays to implementation may have been harmful for public health.

## Teaser

This decision analytical model investigates associations between a federal regulation requiring graphic health warnings on cigarette packaging in the US and changes in estimates of smoking-attributable deaths and life-years gained based on implementation dates of 2012 vs 2022.

## Introduction

Cigarette health warnings in the US have remained unchanged for the past 35 years, but starting in 2022, pending legal challenges, the US Food and Drug Administration (FDA) will require cigarette manufacturers to display graphic health warnings covering 50% of the front and back of cigarette packages. These warnings will also be required in cigarette advertisements. Cigarette health warnings will feature 1 of 13 graphic images that depict some of the negative health consequences of smoking and brief warning statements (eg, “Smoking causes COPD, a lung disease that can be fatal”).^1^ The FDA originally planned to implement graphic health warnings in 2012, but tobacco industry litigation and resulting court decisions struck down the initial warnings, delaying implementation.^1^ Public health organizations and physicians filed a lawsuit against the FDA for these delays, and a court-ordered deadline forced the FDA to finalize new graphic health warnings nearly a decade later.^2^ The tobacco industry has also issued repeated legal challenges to the revised warning designs, and the implementation date is contingent on further court rulings.

Previous estimates of the potential population health benefits of graphic health warnings in the US were based on smoking prevalence trends through 2016.^3^ Since then, the prevalence of smoking—especially among youths and young adults—has decreased more than expected.^4,5^ Recent shifts in smoking trends may affect the outcomes associated with new regulations, including graphic health warnings. Past estimates also have not separately evaluated the outcomes for specific birth cohorts. This study used a simulation model to estimate smoking prevalence and mortality outcomes associated with graphic health warnings in the US under the planned policy for 2022 and compared these with estimated outcomes if the policy had been implemented in 2012.

## Methods

### Smoking History Generator Population Model

This decision analytical model simulated smoking and mortality outcomes associated with graphic health warnings in the US using the Cancer Intervention and Surveillance Modeling Network (CISNET) Smoking History Generator Population Model, a discrete deterministic compartmental model version of the CISNET Smoking History Generator microsimulation model.^6,7,8,9^ The model simulated the annual number of never smokers, current smokers, and former smokers in the US by age (0-99 years), gender (male, female), birth cohort (1864-2100), and calendar year (1964-2100). Former smokers were further categorized by the number of years since quitting smoking (from 1 to 40 or more). Age, birth cohort, and calendar year were modeled in 1-year increments. A schematic representation of the model is shown in the eFigure 1 in the Supplement. In the model, simulated never smokers became current smokers and current smokers became former smokers based on annual smoking initiation and cessation probabilities. Further details are provided in the eAppendix in the Supplement. Data were analyzed from October 2020 to July 2021. This study did not involve human participants or private information and was therefore exempt from institutional review board review.

### Smoking Initiation and Cessation

Annual probabilities of smoking initiation and cessation by age, gender, calendar year, and birth cohort were derived from the CISNET Lung Working Group using National Health Interview Surveys from 1965 to 2018.^8,10^ Current smoking was defined as having smoked at least 100 cigarettes in a lifetime and having smoked at any time in the past 2 years. This stringent definition of current smoking avoided the need to model relapse among individuals who had recently quit smoking but produced smoking prevalence estimates that were slightly greater than standard survey estimates.^11^ Annual probabilities for the birth cohorts beginning with the cohort born in 1864 were derived using age-period-cohort models of National Health Interview Surveys data on smoking initiation and cessation and on the prevalence of never, current, and former smoking status.^8,9,10^ In the baseline scenario, probabilities of initiating smoking were projected into the future by holding them fixed at the estimated level for the 2000 birth cohort (ie, individuals aged 18 years at the last survey in 2018). Analogously, probabilities of smoking cessation were projected into the future by holding these fixed at the value estimated for the 1990 birth cohort (ie, individuals aged 28 years at the last survey in 2018).^8,9^

### Mortality

Population dynamics were based on US Census birth estimates and on mortality probabilities generated by the CISNET Lung Working Group that varied by age, gender, birth cohort, and smoking status (never, current, or former smoker by years since quitting).^8,12,13^ Future mortality rates by cohort, age, and gender were projected using the Lee-Carter method and then partitioned into mortality rates for never, current, and former smokers using the approach of Rosenberg et al.^12,13,14^

For mortality among former smokers, we used probabilities as a function of age (*a*), year (*y*), and years since quitting (*ysq*) based on the CISNET probabilities of mortality among never and current smokers. Specifically, the probability of mortality for a former smoker (*u_fs_*) was calculated by taking the maximum between the probability of mortality for a never smoker (*u_ns_*) of the same age and year and the probability of mortality for a current smoker (*u_cs_*) of the same age and year multiplied by the relative risk (RR) of mortality for a former smoker compared with mortality for current smoker by years since quitting (*RR_fs_*): *u_fs_*(*a,y,ysq*)* = max* (*u_cs_*[*a,y*]* × RR_fs_*[*ysq*], *u_ns_*[*a,y*]).

Estimates of the RR of all-cause mortality among former smokers compared with current smokers by the years-since-quitting categories were derived from Thun et al.^15^ We transformed these into single-year estimates by taking the midpoint of each years-since-quitting category and interpolating it using an exponential model, constraining the (*RR_fs_*) to be between 0 and 1 (eTable 1 and eFigures 2 and 3 in the Supplement). This approach ensured that the probability of mortality for a former smoker (*u_fs_*) was between those for a never smoker (*u_ns_*) and current smoker (*u_cs_*). The relative risk of mortality among former smokers who quit more than 40 years ago was held constant at the relative risk among former smokers who quit 40 years ago.

### Policy Parameters

We modeled (1) a baseline scenario without graphic health warnings, (2) graphic health warning policy effects that modified underlying initiation and cessation probabilities based on a warning implementation date of January 1, 2022, and (3) graphic health warning policy effects if the warnings had been implemented on January 1, 2012. The policy effect sizes were based on estimates developed by previous researchers^3,9^ and were consistent with those of other studies.^16,17,18,19^ However, because policies in the Smoking History Generator act through changes in smoking initiation and cessation and Levy et al^3,9^ reported the outcomes for smoking initiation and prevalence, we further calibrated the model to translate the prevalence effects from Levy et al into changes in cessation probabilities. Our main policy scenario decreased initiation by 10% and increased cessation by 50%. Whereas the association of graphic health warnings with smoking initiation was treated as constant over time, we used a decay rate for cessation that reflected most smokers quitting in response to the policy within the first few years after its implementation. Specifically, the cessation effect was applied with 2012 or 2022 as year 0 (*y0*), with a decay rate of 20% in subsequent years (eg, 50% × [1 − 0.2]*^year^*^−^*^y0^*). The decay rate of 20% was based on prior calibration of the CISNET Smoking History Generator microsimulation model in the evaluation of the effects of cigarette taxes on smoking prevalence and was similarly used by the validated SimSmoke model.^9^

We explored a range of policy scenarios in which graphic health warnings reduced smoking initiation across all ages by 5% to 15% and increased cessation across all ages by 25% to 75% (9 scenarios in 2 policy implementation years). In addition, we evaluated a maximum smoking-reduction scenario in which all smoking stopped by the end of 2022, with no new smoking initiation and complete cessation among all current smokers. This estimated the largest possible public health gains that could be achieved through intervention, because smoking-attributable deaths among existing former smokers cannot be avoided with further intervention.^20^

### Modeling Practices

When possible, we adhered to recommendations for best research practices in simulation modeling.^21,22^ We took a societal perspective to simulate the US population over a long time with annual cycle lengths and used a model structure consistent with existing smoking models.^23,24^ Our state-transition model simulated US cohorts using transition probabilities for smoking that were specific to the historical patterns of each birth cohort. These smoking initiation and cessation transition probabilities were the most representative data sources for our purposes and are among the most widely used in the field of tobacco simulation modeling. Although the model maintained the Markov property, former smokers were distinguished by time since quitting, which allowed the model to simulate changes in their mortality risk depending on how recently they stopped smoking. For intervention effects, existing literature was challenging to translate into model-ready inputs. Therefore, we relied on initiation and cessation effect size estimates determined by experts on cigarette graphic health warnings, which were used in a previous analysis.^3^ Because a policy on graphic health warnings for cigarettes has never been implemented in the US, we were unable to perform external validation for our policy scenarios; however, the model successfully reproduced observed smoking patterns using the baseline scenario. To facilitate transparency, the CISNET initiation, cessation, and mortality inputs are publicly available online.^25^ Model equations are provided in the eAppendix in the Supplement.

### Statistical Analysis

For each scenario, we used methods similar to those of Tam et al^9^ to estimate smoking prevalence, smoking-attributable deaths averted, and life-years gained from policy implementation to 2100 in the US population. In brief, smoking-attributable deaths (SAD) were calculated using the Equation:
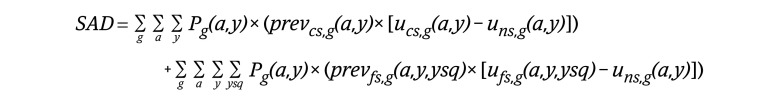
where for a given gender (*g*), age (*a*), and year (*y*), the prevalence of current smoking (*prev_cs,g_*(*a,y*)) was multiplied by the difference in mortality rate between a current smoker (*u_cs,g_*(*a,y*)) and never smoker (*u_ns,g_*(*a,y*)) and then applied to the size of the population (*P_g_*(*a,y*)) at that gender, age, and year to produce the estimated number of smoking-attributable deaths associated with current smoking. This was summed with the number of corresponding smoking-attributable deaths associated with former smoking to produce the total estimated number of smoking-attributable deaths.

The estimated number of smoking-attributable deaths averted in each year was calculated by subtracting the difference in deaths between policy and baseline scenarios across all ages. To calculate the number of life-years gained, we calculated the difference in the total number of life-years lived in the baseline scenario and each policy scenario. All calculations were performed using R, version 4.0.4 (R Project for Statistical Computing).

## Results

**Figure 1.**
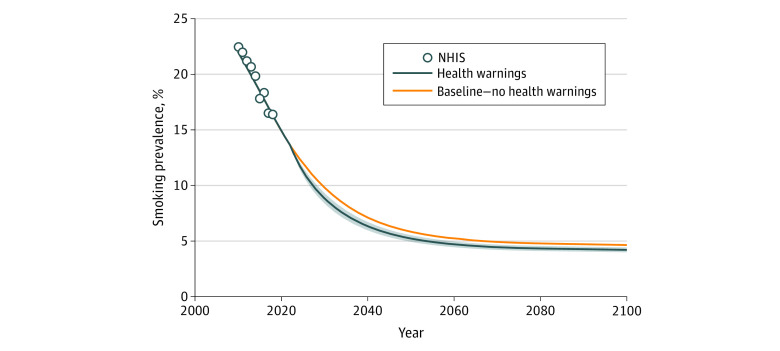
Estimated Annual Prevalence of Smoking in the US Using the Baseline and Graphic Health Warning Scenarios, 2022 to 2100 Current smokers include individuals who had smoked within the past 2 years and recently quit. Shading represents the ranges of the scenarios. NHIS indicates National Health Interview Surveys.

The model reproduced observed US smoking trends from 2010 to 2018, projecting that smoking prevalence would reach 5% before 2060 in the baseline scenario (Figure 1). In the baseline scenario, the model estimated that smoking prevalence would decrease from 20.5% in 2012 to 13.6% in 2022 and 4.6% in 2100. Simulated implementation of graphic health warnings in both 2022 and 2012 was associated with modest reductions in estimated smoking prevalence compared with the baseline scenario. In the scenario with graphic health warnings implemented in 2022, the model estimated that smoking prevalence would decrease from 13.6% in 2022 to 4.2% (range across policy scenarios, 4.0%-4.4%) in 2100. If the warnings had been implemented in 2012, the model estimated that smoking prevalence would have decreased to 12.3% (range, 11.7%-12.9%) in 2022 and 4.2% (range, 4.0%-4.4%) in 2100.

In the baseline scenario, the model estimated 13.2 million smoking-attributable deaths from 2012 to 2100, with 8.8 million among men and 4.4 million among women. In the maximum smoking-reduction scenario, in which all smoking ceased by 2022, the model estimated that 5.5 million smoking-attributable deaths would be avoided (men, 3.3 million; women, 2.1 million) through 2100. The total estimated number of life-years gained would be 81.8 million (men, 52.1 million; women, 29.8 million). The full range of estimates for cumulative smoking-attributable deaths averted and life-years gained by 2100 based on the scenarios of policy implementation in 2012 and 2022 are presented in the Table (cumulative results by 2032, 2042, and 2052 are shown in eTables 2-4 in the Supplement).

**Table.** Estimated Cumulative Smoking-Attributable Deaths Averted and Life-Years Gained From 2012 to 2100 in the US

Increase in smoking cessation^a^	Reduction in smoking initiation					
	Policy implemented in 2022			Policy implemented in 2012		
	5%	10%	15%	5%	10%	15%
Smoking-attributable deaths averted, thousands						
25%	275^b^	333	392	365^b^	451	539
50%	481	539^c^	598	632	718^c^	805
75%	678	735	794^d^	888	973	1060^d^
Life-years gained, millions						
25%	4.0^b^	5.0	6.1	5.7^b^	7.4	9.1
50%	6.9	7.9^c^	8.9	9.6	11.2^c^	12.9
75%	9.6	10.6	11.6^d^	13.3	15.0	16.6^d^

^a^ The cessation effect was applied for the scenarios with graphic health warnings implemented in 2012 or 2022 as year 0 (*y0*) with a decay rate of 20% in subsequent years.

^b^ Most conservative scenario.

^c^ Main estimate scenario.

^d^ Most optimistic scenario.

**Figure 2.**
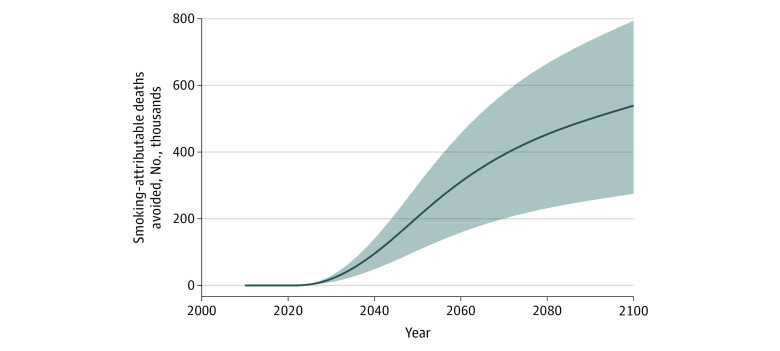
Cumulative Smoking-Attributable Deaths Averted in the US Using a Graphic Health Warning Scenario, 2022 to 2100 The solid line represents the main policy scenario (initiation reduced by 10% and cessation increased by 50% in 2022). Shading represents the most optimistic (initiation reduced by 15% and cessation increased by 75% in 2022) and conservative (initiation reduced by 5% and cessation increased by 25% in 2022) scenarios.

**Figure 3.**
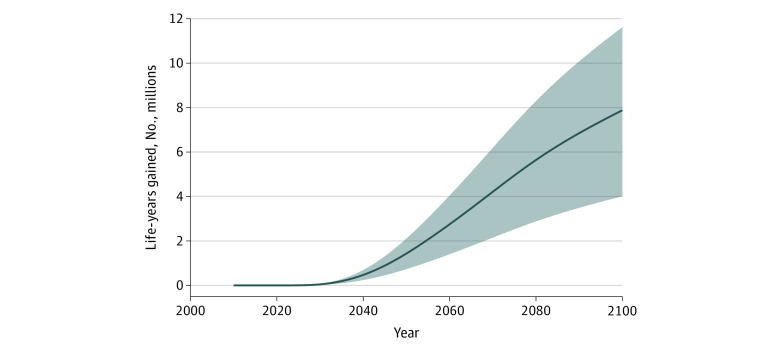
Cumulative Life-Years Gained in the US Using a US Graphic Health Warning Scenario, 2022 to 2100 The solid line represents the main policy scenario (initiation reduced by 10% and cessation increased by 50% in 2022). Shading indicates the most optimistic (initiation reduced by 15% and cessation increased by 75% in 2022) and conservative (initiation reduced by 5% and cessation increased by 25% in 2022) scenarios.

In the main policy scenario, implementation in 2022 would be associated with an estimated 539 000 (range, 275 000-794 000) smoking-attributable deaths averted (Figure 2) and 7.9 million (range, 4.0-11.6 million) life-years gained (Figure 3) from 2022 to 2100 and would avert 4.1% (range, 2.1%-6.0%) of the estimated number of smoking-attributable deaths in the baseline scenario. In the scenario with a graphic health warnings policy implemented in 2022, 9.9% (range, 5.0%-14.6%) of the total number of smoking-attributable deaths would be averted and 9.6% (range, 4.9%-14.2%) of the life-years would be gained compared with the maximum smoking reduction scenario.

If graphic health warnings had been implemented in 2012, the model estimated that policy implementation would have been associated with 33.2% (range, 32.9%-33.5%) more smoking-attributable deaths averted (718 000; range, 365 000 to 1.1 million) and 42.7% (range, 42.3%-43.1%) more life-years gained (11.2 million; range, 5.7-16.6 million) compared with estimates based on implementation in 2022. A total of 5.4% (range, 2.8%-8.0%) of the estimated number of smoking-attributable deaths in the baseline scenario from 2012 to 2100 would have been averted.

## Discussion

In this decision analytical model, implementation of graphic health warnings was associated with reductions in estimated smoking-related mortality. This was consistent with earlier estimates in the US^3^ and other countries.^26^ The reductions in overall estimated smoking prevalence were similar to those reported in Levy et al^3^: 10% (range, 4%-19%). However, this study’s model estimated fewer smoking-attributable deaths averted during a longer period owing to the model’s projections of lower smoking prevalence in the baseline scenario compared with the projections of Levy et al.^3^ Recent studies have found that smoking rates among US youths and young adults have decreased at especially fast rates^4,5^; thus, the outcomes of future policy changes will be attenuated by these underlying trends at baseline.

This model found that implementing graphic health warnings in 2012, as originally planned based on the FDA’s 2011 rule, may have been associated with 33.2% more smoking-attributable deaths averted and 42.7% more life-years gained, suggesting potential health consequences associated with delayed implementation and tobacco industry litigation. Future legal challenges to FDA tobacco regulations may be associated with substantial harms to population health and should also be quantified.

We acknowledge that the counterfactual of having had no delays to the implementation of graphic health warnings in 2012 is unlikely to reflect real-world policy making. Past policy delays cannot be undone, and protracted litigation in the present tobacco regulatory environment may be inevitable. Other factors beyond legal challenges, such as limited agency capacity (eg, resources and personnel) and changing leadership, can delay the implementation of regulations. In some instances, such as a lawsuit filed against the FDA by public health organizations, a court decision may expedite policy implementation. However, by quantifying the potential losses associated with delayed implementation, the findings of this study suggest a need to act quickly while minimizing the chances of future procedural delays.

Only 4% of all estimated smoking-attributable deaths were averted in this study’s scenario of implementing a graphic health warnings policy in 2022. Furthermore, the model estimated that graphic health warnings implemented from 2022 to 2100 would be associated with 9.9% of the total number of smoking-attributable deaths avoided compared with the maximum smoking-reduction scenario. This finding suggests the need for progressive policy action that goes beyond graphic health warnings. The Biden Administration’s recent commitment to implement a federal ban on menthol flavoring in combustible cigarettes may have positive implications for public health.^27^ Even larger health gains would likely be achieved by reducing the level of nicotine in combustible cigarettes to minimally addictive levels.^28^

We believe that rigorous evaluations of graphic health warnings using state-of-the-art methods are needed to accurately quantify their outcomes. Future research should carefully evaluate the effects of the planned graphic health warnings after implementation and pay particular attention to changes to smoking behaviors in response to the policy, including smoking cessation, reduced smoking intensity, switching to noncigarette tobacco products, smoking initiation, and youth experimentation with smoking. Furthermore, researchers should consider how multiple factors, including e-cigarette use and other tobacco control policies, could confound estimates of policy outcomes.

### Limitations

This study has limitations. Although the literature on graphic health warnings reports an associated public health benefit,^16,17,19,29,30,31,32,33^ uncertainty remains about the true magnitude of their effect on smoking behavior, especially with regard to smoking initiation.^34^ To our knowledge, the most rigorous analysis conducted to date evaluated the graphic health warnings policy in Canada using the US as a control.^35^ The current study estimated changes to overall smoking prevalence and not smoking behaviors per se, similar to other analyses.^19,35^ We modeled smoking through initiation and cessation behaviors, which required inputs that were challenging to produce using existing empirical methods. Even in studies with appropriate controls, causality cannot be evaluated because graphic health warnings can be implemented at the same time as other policies. For these reasons, we structured this study as an uncertainty analysis by evaluating policy effects under both optimistic and conservative policy conditions using previously reported upper and lower threshold values based on experts’ opinion of the likely association of graphic health warnings with smoking initiation and cessation. Although initiation effects are understudied,^34^ other research on cessation effects was consistent with our estimates.^18,19,36^

We also did not consider potential associations between graphic health warnings and the quantity of cigarettes consumed by individual smokers. We did not find substantial evidence in the literature to justify such associations. Although the quantity of cigarettes smoked may decrease for those who continue to smoke after the implementation of such a policy, the mean overall quantity of cigarettes smoked may increase if those who quit as a result of a graphic health warning policy smoked less intensely than did the remaining smokers.

We did not present uncertainty analyses in our baseline projections. Although this could influence our baseline scenario results in terms of absolute numbers, this study focused on the health gains associated with policies. Any uncertainty in the underlying baseline model inputs (all-cause mortality rates and CISNET initiation and cessation estimates) would carry through to the policy scenario, and relative changes would remain qualitatively the same.

The effects estimated by the model are based on the CISNET projections of smoking rates using data through 2018. We did not account for changes in smoking or changes in underlying mortality as a result of the COVID-19 pandemic. The health benefits associated with graphic health warnings will depend on a range of factors not considered in this model, including tobacco control policies, industry activities, and the use of other tobacco and nicotine products such as e-cigarettes. In addition, we did not explicitly consider e-cigarettes in the model. However, the underlying CISNET smoking parameters captured recent changes in smoking initiation and cessation through 2018 and cohort effects that may reflect recent e-cigarette use. Graphic health warnings proposed by the FDA in 2011 differ from those planned for 2022, but this study assumed that the association of the warnings with smoking behaviors would be the same.

Although this model’s results were consistent with earlier estimates using the SimSmoke model,^3^ they differed in absolute terms partly because the current model did not explicitly incorporate the effects of past policies. Furthermore, the projections accounted for more recent decreases in the prevalence of smoking by birth cohort, which resulted in a lower projected baseline smoking prevalence in the absence of graphic health warnings.

## Conclusions

This decision analytical model estimated that FDA cigarette graphic health warnings, if implemented in 2022, would be associated with public health benefits. The model also estimated that more smoking-attributable deaths would have been averted if the policy had been implemented in 2012. This study’s findings suggest that preventing delays to policy implementation should be a public health priority.

## Notes

Supplement.
eAppendix.
**eFigure 1. **CISNET Smoking History Generator Population Model Diagram **eTable 1.** Former Smoker Relative Risk of Mortality by Years Since Quit Vs Current Smoker **eFigure 2.** Former Smoker Exponential Model Interpolation of Relative Risk of Mortality by Years Since Quit Vs Current Smoker, Men **eFigure 3.** Former Smoker Exponential Model Interpolation of Relative Risk of Mortality by Years Since Quit Vs Current Smoker, Women **eTable 2.** Predicted Cumulative Smoking Attributable Deaths Averted (In Thousands) and Life-Years Gained (In Millions) From 2012-2032 **eTable 3.** Predicted Cumulative Smoking Attributable Deaths Averted (In Thousands) and Life-Years Gained (In Millions) From 2012-2042 **eTable 4.** Predicted Cumulative Smoking Attributable Deaths Averted (In Thousands) and Life-Years Gained (In Millions) From 2012-2052
