# Supplementary material for: Estimated Prevalence of Smoking and Smoking-Attributable Mortality Associated With Graphic Health Warnings on Cigarette Packages in the US From 2022 to 2100
Source: JAMA Health Forum. 2021 Sep 24;2(9):e212852. doi: 10.1001/jamahealthforum.2021.2852 (PMC8796938; doi:10.1001/jamahealthforum.2021.2852)
Supplement: Supplement. — eAppendix. eFigure 1. CISNET Smoking History Generator Population Model Diagram eTable 1. Former Smoker Relative Risk of Mortality by Years Since Quit Vs Current Smoker eFigure 2. Former Smoker Exponential Model Interpolation of Relative Risk of Mortality by Years Since Quit Vs Current Smoker, Men eFigure 3. Former Smoker Exponential Model Interpolation of Relative Risk of Mortality by Years Since Quit Vs Current Smoker, Women eTable 2. Predicted Cumulative Smoking Attributable Deaths Averted (In Thousands) and Life-Years Gained (In Millions) From 2012-2032 eTable 3. Predicted Cumulative Smoking Attributable Deaths Averted (In Thousands) and Life-Years Gained (In Millions) From 2012-2042 eTable 4. Predicted Cumulative Smoking Attributable Deaths Averted (In Thousands) and Life-Years Gained (In Millions) From 2012-2052 [file jamahealthforum-e212852-s001.pdf]

Supplementary Online Content

Tam J, Jeon J, Thrasher JF, et al. Estimated prevalence of smoking and smoking-attributable mortality associated with graphic health warnings on cigarette packages in the US from 2022 to 2100. *JAMA Health Forum*. 2021;2(9):e212852. doi:10.1001/jamahealthforum.2021.2852

eAppendix.
eFigure 1. CISNET Smoking History Generator Population Model diagram
eTable 1. Former smoker relative risk of mortality by years since quit vs current smoker
eFigure 2. Former smoker exponential model interpolation of relative risk of mortality by years since quit vs current smoker, men
eFigure 3. Former smoker exponential model interpolation of relative risk of mortality by years since quit vs current smoker, women
eTable 2. Predicted cumulative smoking attributable deaths averted (in thousands) and life-years gained (in millions) from 2012-2032
eTable 3. Predicted cumulative smoking attributable deaths averted (in thousands) and life-years gained (in millions) from 2012-2042
eTable 4. Predicted cumulative smoking attributable deaths averted (in thousands) and life-years gained (in millions) from 2012-2052

This supplementary material has been provided by the authors to give readers additional information about their work.

© 2021 Tam J et al. *JAMA Health Forum*.

eFigure 1. CISNET Smoking History Generator Population Model Diagram

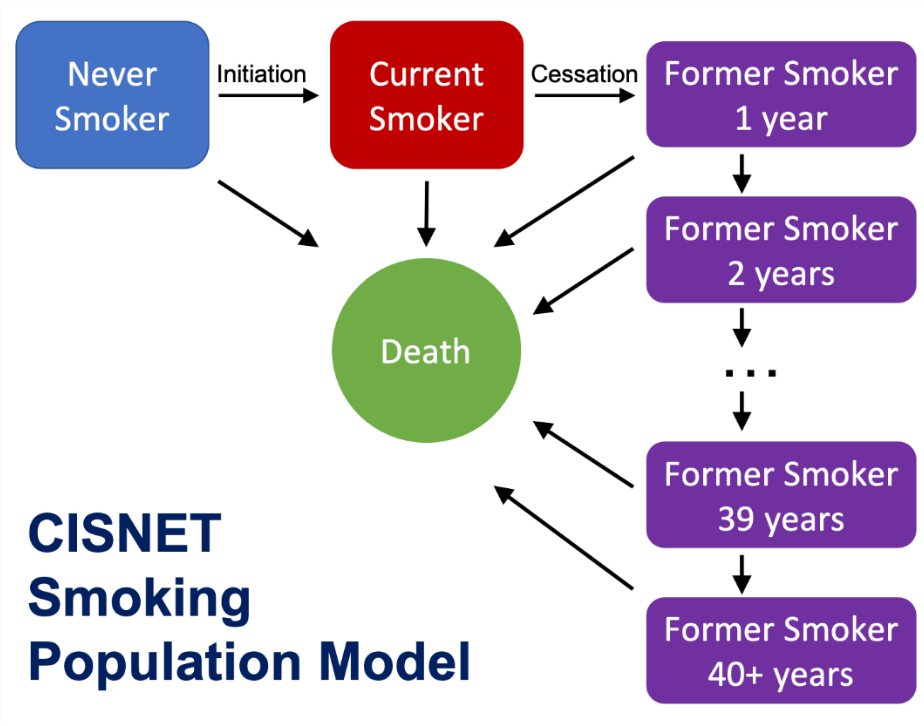

Model equations

For each gender and birth cohort *c = 1864 to 2100*, the model tracks the number of individuals in each of the smoking status compartments *Never[a,c]*, *Current[a,c]*, *Former[a,c,j]*, where *a = 0 to 99* denotes age and *j = 1 to 40*. Here a and j denote age and years since quit for former smokers, respectively. Individuals born in year *c* are all assigned to the never smoker compartment at age *a=0*, where *Births[c]* denote the number of births in year c. Let *Never[a,c]* and *Current[a.c]* be the number of never smokers and current smokers at age a who were born in year c. Similarly, let *Former[a,c,j]* be the number of former smokers at age a who were born in year c and quit smoking for j years. Individuals then move between compartments annually according to the smoking initiation (*init[a,c]*), cessation (*cess[a,c]*) and death probabilities by smoking status (*deathnever[a,c]*, *deathcurrent[a,c]*, *deathformer[a,c,j]*) and the following equations:

Loop over birth-cohorts
For (c=1864 to 2100)

$Never[0, c] = Births[c]$
$Current[0, c] = 0$
$Former[0, c, j] = 0, j = 1, \ldots, 40$

© 2021 Tam J et al. *JAMA Health Forum*.

Loop over ages
For (a=0 to 99)

$$Never[a,c] = Never[a-1,c] * (1 - init[a-1,c]) * (1 - deathnever[a-1,c])$$

$$Current[a,c] = Never[a-1,c] * init[a-1,c] * (1 - deathnever[a-1,c]) + Current[a-1] * (1 - cess[a-1,c]) * (1 - deathcurrent[a-1,c])$$

$$Former[a,c,1] = Current[a-1,c] * cess[a-1,c] * (1 - deathcurrent[a-1,c])$$

Loop over years since quit
For (j=2:39)

$$Former[a,c,j] = Former[a-1,c,j-1] * (1 - deathformer[a-1,c,j-1])$$

$$Former[a,c,40] = Former[a-1,c,39] * (1 - deathformer[a-1,c,39]) + Former[a-1,c,40] * (1 - deathformer[a-1,c,40])$$

eTable 1. Former smoker relative risk of mortality by years since quit vs. current smoker

Years since quitting	Mid-point	Men - RR former vs current smoker	Women - RR former vs current smoker
<2	1	1.07*	0.87
2-4	3	1.02*	0.75
5-9	7	0.83	0.84
10-19	14.5	0.77	0.55
20-29	24.5	0.54	0.49
30-39	34.5	0.47	0.41

* Not statistically significantly different from 1, so set to 1 for development of RR model.
RR = relative risk estimates. See Thun MJ, Carter BD, Feskanich D, et al. 50-year trends in smoking-related mortality in the United States. *N Engl J Med.* 2013;368(4):351-364.

© 2021 Tam J et al. *JAMA Health Forum*.

eFigure 2. Former smoker exponential model interpolation of relative risk of mortality by years since quit vs. current smoker, Men

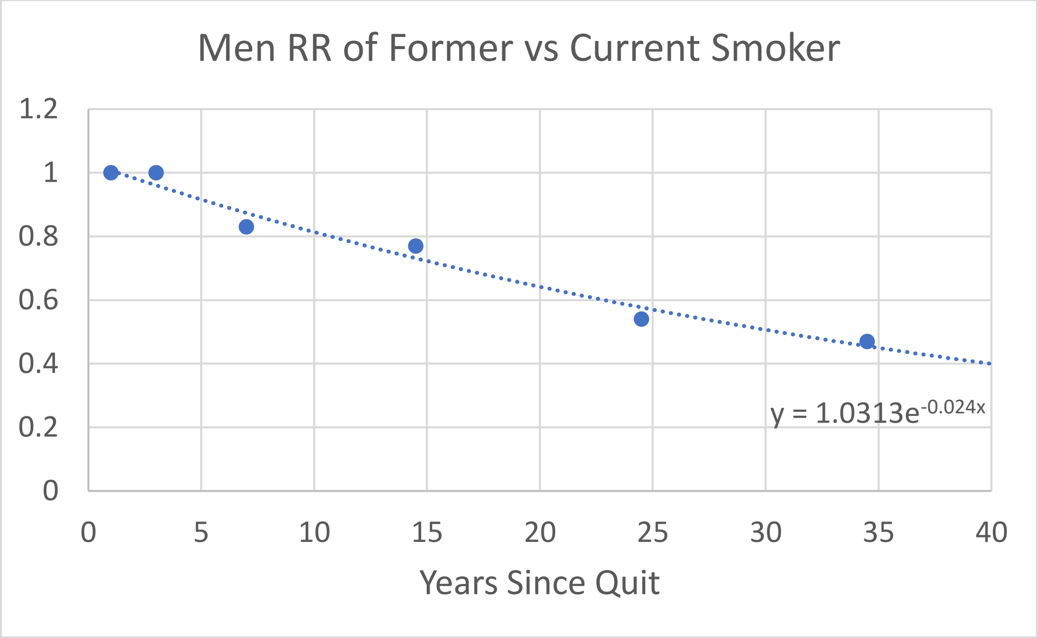

eFigure 3. Former smoker exponential model interpolation of relative risk of mortality by years since quit vs. current smoker, Women

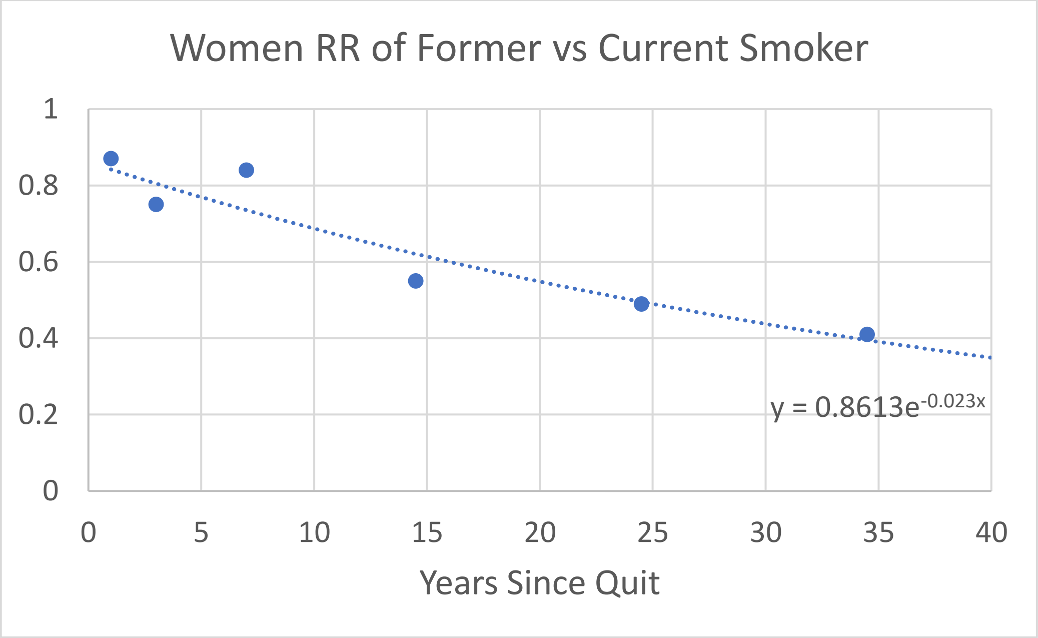

© 2021 Tam J et al. *JAMA Health Forum*.

eTable 2. Predicted Cumulative Smoking Attributable Deaths Averted (in thousands) and Life-Years Gained (in millions) from 2012-2032

	Policy implemented in 2022			Policy implemented in 2012		
	Smoking initiation reduced by			Smoking initiation reduced by		
Smoking cessation increased by[a]	5%	10%	15%	5%	10%	15%
	Smoking-attributable deaths averted (thousands)			Smoking-attributable deaths averted (thousands)		
25%	**15.7** [b]	15.9	16.0	**67.0** [b]	68.0	68.9
50%	30.7	**30.9** [c]	31.0	130.5	**131.5** [c]	132.4
75%	45.2	45.3	**45.4** [d]	191.5	192.5	**193.4** [d]
	Life-years gained (millions)			Life-years gained (millions)		
25%	**0.05** [b]	0.05	0.05	**0.35** [b]	0.36	0.36
50%	0.09	**0.09** [c]	0.09	0.69	**0.69** [c]	0.70
75%	0.13	0.13	**0.13** [d]	1.01	1.01	**1.02** [d]

[a] Cessation effect is applied in 2012 or 2022 (y0) with a decay rate of 20% in subsequent years (e.g. $50\% \times (1 - 0.2)^{year-(y0-1)}$).
[b] "Most conservative" scenario.
[c] "Main estimate" scenario.
[d] "Most optimistic" scenario.

© 2021 Tam J et al. *JAMA Health Forum*.

eTable 3. Predicted Cumulative Smoking Attributable Deaths Averted (in thousands) and Life-Years Gained (in millions) from 2012-2042

	Policy implemented in 2022			Policy implemented in 2012		
	Smoking initiation reduced by			Smoking initiation reduced by		
Smoking cessation increased by[a]	5%	10%	15%	5%	10%	15%
	Smoking-attributable deaths averted (thousands)			Smoking-attributable deaths averted (thousands)		
25%	**59.2** [b]	59.9	60.7	**141.7** [b]	145.3	148.8
50%	115.0	**115.8** [c]	116.5	274.4	**277.9** [c]	281.4
75%	168.5	169.2	**170.0** [d]	401.9	405.3	**408.8** [d]
	Life-years gained (millions)			Life-years gained (millions)		
25%	**0.32** [b]	0.32	0.32	**1.04** [b]	1.06	1.08
50%	0.62	**0.62** [c]	0.63	2.01	**2.04** [c]	2.06
75%	0.91	0.91	**0.91** [d]	2.95	2.97	**3.00** [d]

[a] Cessation effect is applied in 2012 or 2022 (y0) with a decay rate of 20% in subsequent years (e.g. $50\% \times (1-0.2)^{year-(y0-1)}$).
[b] "Most conservative" scenario.
[c] "Main estimate" scenario.
[d] "Most optimistic" scenario.

© 2021 Tam J et al. *JAMA Health Forum*.

eTable 4. Predicted Cumulative Smoking Attributable Deaths Averted (in thousands) and Life-Years Gained (in millions) from 2012-2052

	Policy implemented in 2022			Policy implemented in 2012		
	Smoking initiation reduced by			Smoking initiation reduced by		
Smoking cessation increased by[a]	5%	10%	15%	5%	10%	15%
	Smoking-attributable deaths averted (thousands)			Smoking-attributable deaths averted (thousands)		
25%	**116.6** [b]	119.2	121.9	**213.7** [b]	223.4	233.2
50%	225.4	**228.1** [c]	230.7	409.4	**419.0** [c]	428.6
75%	329.5	332.1	**334.7** [d]	597.1	606.6	**616.1** [d]
	Life-years gained (millions)			Life-years gained (millions)		
25%	**0.86** [b]	0.87	0.89	**2.01** [b]	2.09	2.17
50%	1.66	**1.68** [c]	1.70	3.86	**3.94** [c]	4.02
75%	2.43	2.45	**2.47** [d]	5.65	5.72	**5.80** [d]

[a] Cessation effect is applied in 2012 or 2022 (y0) with a decay rate of 20% in subsequent years (e.g. $50\% \times (1-0.2)^{year-(y0-1)}$).
[b] "Most conservative" scenario.
[c] "Main estimate" scenario.
[d] "Most optimistic" scenario.

© 2021 Tam J et al. *JAMA Health Forum*.
